# Bacteriological water quality in school’s drinking fountains and detection antibiotic resistance genes

**DOI:** 10.1186/s12941-016-0176-7

**Published:** 2017-02-08

**Authors:** Denize Gomes Freitas, Rassan Dyego Romão Silva, Luis Artur Mendes Bataus, Mônica Santiago Barbosa, Carla Afonso da Silva Bitencourt Braga, Lilian Carla Carneiro

**Affiliations:** 1 Biology Department, State University of Goiás UEG, Morrinhos, Brazil; 2 Biophysic Department, Federal University of Goiás, Goiânia, Brazil; 3 IPTSP Department, Federal University of Goiás, Goiânia, Brazil; 4 235 Street, Leste Universitário Neighborhood, Goiânia, GO 74605-050 Brazil

**Keywords:** Microorganism, Plasmid stability, Bacterial conjugation, Contaminants, Water, Health

## Abstract

The fecal coliform can contaminate water of human consumption causing problems to public health. Many of these microorganisms may contain plasmid and transfer them to other bacteria. This genetic material may confer selective advantages, among them resistance to antibiotics. The objectives of this study were to analyze the presence of fecal coliforms in water and at drinker surface, to identify the existence of plasmid, conducting studies of resistance to antibiotics, plasmid stability and capacity of bacterial conjugation. Were collected microorganisms in water of drinker surface and were used specific culture media and biochemical tests for identification of organisms, tests were performed by checking the resistance to antibiotics (ampicillin 10 μg, tetracycline 30 μg, and ciprofloxacin 5 μg), was performed extraction of plasmid DNA, plasmid stability and bacterial conjugation. Was obtained results of 31% of *Salmonella* spp. and 51% for other coliforms. Among the samples positive for coliforms, 27 had plasmid stable and with the ability to perform conjugation. The plasmids had similar forms, suggesting that the resistance in some bacteria may be linked to those genes extra chromosomal.

## Background

Water is essential to life, but many people do not have access to clean and safe drinking water and many die of waterborne bacterial infections. Understanding water quality is vital to worldwide public health. The access to potable water increases the lifespan and improves the health of world citizens. Several diseases can be provoked by pathogenic microorganisms found in contaminated water [1]. The Ministry of Health (Ministério da Saúde 200) by ordinance 518, resolution number 20 of 1986 of CONAMA (National Council for the Environment), provides that water for human consumption must be free of microorganisms having a patterning acceptable with values of 200 CFU/100 mL, 1000 un/100 mL and 4000 un/100 mL for thermo tolerant coliforms.

The human activity produce contamination in water, the majority of microorganisms presents are coliforms, which indicates human or animal fecal contamination of water [2, 3].

*Escherichia coli*, *Klebsiella* sp. and *Salmonella* sp. are enterobacteria frequently isolated from human biological materials. Several outbreaks of food poisoning are associated with poor sanitation conditions such as failure of hand hygiene or bad cleaning [4–6]. Salmonellosis, one of the most commom diseases that can be transmitted by food, is caused by the bacteria salmonella [7–9].

The presence of *Salmonella* sp. in water can endanger human health, due the presence of antibiotic resistance genes [10]. Many antibiotic resistance genes reside on transmissible plasmids.

Important biological functions are associated with plasmids in *Salmonella* including virulence factors, resistance to heavy metals, antibiotics, phages and utilization of carbon sources [10]. The virulence plasmids usually have high molecular weight [11]. The dissemination of the plasmid may cause an increase in bacterial resistance [12], interfering with the clinical importance of infections by contributing to increased spread of antibiotic resistance genes [13].

The aim of this study was to assess the bacteriological water quality in school’s drinking fountains and detection of cryptic plasmids harboring antibiotic resistance genes.

## Methods

### Collection and cultivation of microorganisms

The 110 biological samples used in this study were isolated from water and from drinking fountains surface in public schools of Morrinhos—GO. Were collected 51 samples from the button that controls the output of water, 51 from pipe where the water comes out and eight from water samples. A 10 mL aliquot was aseptically collected from each school visited. The samples from drinking fountains surfaces were obtained using swabs. The samples were cultivated in nutrient broth. After 24 h of incubation, samples were maintained at 4 °C, and peaked monthly.

### Bacterial identification

Were isolated samples from 51 troughs. The coliforms were identified by culture media, using the EC broth (*Escherichia coli*) and SS agar (Salmonella and Shigella media). After procedure the culture tests, the samples were conducted to biochemical tests: glucose [14], lactose [15] and Citrate’s Simons [16].

### Antimicrobial susceptibility

The antibiogram was performed through of methodology for disk of diffusion by Bauer & Kirby, according the criteria established by the National Committee for Clinical Laboratory Standards [17]. The inoculation was circulated through of scan using swab on the surface of the Mueller–Hinton agar, then were placed discs of antibiotics (ampicillin 10 μg, tetracycline 30 μg and ciprofloxacin 5 μg) in agar, with the assistance of sterile forceps. After placing the discs, the plates were inverted and incubated at 36 °C for 24 h, latterly were analyzed through of inhibition halos to ampicillin considered resistant (<14 mm), intermediate (14–16 mm), sensitive (>17 mm); to tetracycline are resistant (<14 mm), intermediate (15–18 mm), sensitive (>19 mm) to ciprofloxacin are resistant (<15 mm), intermediate (16–20 mm), sensitive (>21 mm).

### Plasmid DNA extraction

Based on the manual extraction kit FLEXIPREP of Pharmacia^®^. Bacteria were grown overnight into 5 mL of Luria–Bertani medium and incubated at 37 °C under agitation of 150 rpm. An aliquot of 1.5 mL was separated and processed by extraction of plasmid DNA according manufacturer’s protocol.

### Electrophoresis in agarose gel

According to (Green and Sambrook, [18]) the samples of plasmid DNA were analyzed in agarose gel 1% (p/v) colored with ethidium bromide, dissolved in TEB 0.5× and ethidium bromide (0.2 μg/mL). The gel was subjected to amperage of 30 mA until that sample to enter in the well, and then adjusted to 60 mA. Were visualized the bands of plasmid DNA by ultraviolet radiation of low intensity. Were used a marker of weight molecular of 1 Kb DNA ladder from promega^®^ for comparing the size of the fragments.

### Plasmid stability

(1) The positive samples with cryptic plasmid, were inoculated in nutrient agar, supplemented with ampicillin and incubated at 37 °C overnight. (2) After incubation, 5 µL from cultive, were diluted in 5 mL of nutrient without antibiotic and incubated at 37 °C overnight, after growth, (3) aliquots supplemented with ampicillin and aliquots without ampicillin, were placed on nutrient 1.0% (p/v) and incubated again at 37 °C overnight. The (2 and 3) procedure were repeated four times. The pLK39 plasmid was kindly provided by [19]. The pLK39 plasmid was the standard for comparison with the plasmid studied in this work.

### Conjugation

The conjugation followed the method described by Mitsuhashi et al. [20], with modifications. The donor sample (coliform containing cryptic plasmids) and the receptors with characteristics lac Z negative (*Escherichia coli* DH5α) were cultivation separately on L medium. After 18 h, were mixed in two broths of cultivation, incubated the bacterial cultivation for 24 h and selected the trans conjugants plate ding on LA containing ampicillin and X-Gal chromogenic. After growth, observed the appearance of blue and white colonies.

### Statistical test

We used the statistical test Chi square, SYSTAT 13 to verify if the contamination of local of trough and water were in a high amount of contamination and if was differences between the sites of contamination.

## Results and discussion

Among the 110 samples obtained, 21 were negative (three of water, eight by button that controls the water output and ten of pipes), 89 samples showed microbial growth, (Table 1). Of the water samples from button that control the water output, in five of them were found contamination, however, no one was positive to fecal coliforms. Santana et al. [21], analyzed samples from trough of schools in the city of Brejo da Cruz, PB, and found 26% of the samples contaminated by *Escherichia coli*.

**Table 1:** Profile of bacterial sensitivity antimicrobials tested

Antibiotic	Resistant (%)	Intermediate (%)	Sensitive (%)
Ampicillin	14.6	9	76.4
Tetracycline	7.9	10.1	82
Ciprofloxacin	3.4	–	96.6

In this study, among 89 positive samples (49.44%) showed contamination by fecal coliforms and other 50.56% of the samples were contaminated by other bacteria (data not shown). Analyzing the surface samples of drinkers, found that 50% were positive for fecal coliforms, data similar to those of Silva et al. [22], who found positive for 58.82% of strains isolated from surface of trough. Among the samples classified in this article, 6.67% were *Klebsiella* sp. 12.22% *Escherichia coli* and 31.11% of *Salmonella* sp.

With analyze of the statistical data we conclude that the water contamination not was statistically significant (x^2^ = 2, p = 0.16) in samples collected from the button of trough and of pipe, there was statistical difference of contamination (x^2^ = 26.84, p < 0.0001; x^2^ = 18.84, p < 0.000014). Performing a comparison between the contaminated sites concluded that the contamination rate is equal between the water and button, water and pipe, pipe and button (respectively, x^2^ = 0.09, p = 0.77; x^2^ = 0.01, p = 0.90; x^2^ = 0.28, p = 0.59).

Were performed tests of antibiogram in all samples and 85% them were sensitive to antibiotics (ampicillin, tetracycline and ciprofloxacin). The profile of bacterial sensitivity antimicrobials tested are 14.6% resistant to ampicillin, 7.9% resistant to tetracycline and 3.4% resistant to ciprofloxacin. The intermediate results show 9% to ampicillin and 10.1% to tetracycline.

The Gram-positive bacteria found are highly sensitive to penicillin and sulfonamides, as well as anionic detergents and resistance to sodium azide [23]. When analyzing the plasmids types, the interpretation of results obtained shown that the plasmids obtained are around 10 Kb.

**Fig. 1:**
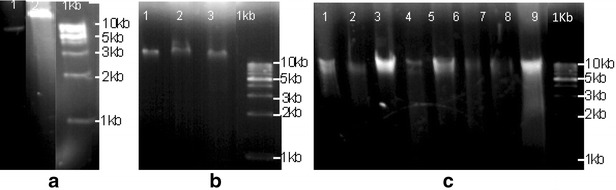
Plasmidial DNA profile. The bands on **a**, **b** and **c** represent the PCR amplification of plasmidial DNA profile

Studies developed by Akturk et al. [24], in fountain drinking water, showed contamination by *Proteus vulgaris*, *Escherichia coli*, *Pseudomonas aeruginosa* and *Citrobacter* spp. The antimicrobial susceptibility study, showed resistance to bacitracin, vancomycine, cephalothin and ampicillin. Plasmid DNA was isolated from 22 isolates; some of them contain a high-molecular weight plasmid DNA. Similar results were found in this study, where bacteria from fountain drinking water presented plasmid with molecular weight more than 10 Kb. Based in this information’s, suggest that the antibiotic resistance, found on bacteria isolated in this work are from plasmids (Fig. 1).

After to isolate and to characterize the plasmids, were done a plasmidial preparation to assess cryptic plasmid stability from coliforms samples found in this work. This information is important to certify if the bacteria that have the plasmid, have possibility of confer antibiotic resistance to future generations.

**Fig. 2:**
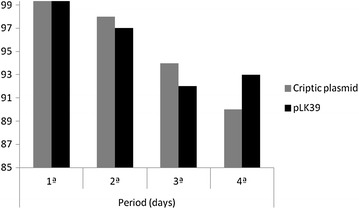
Analysis of the stability of plasmids found in fecal coliforms

The experiments were carried together with another plasmid (pLK39 containing the kanamycin resistance gene) and remained stable in cells of *E. coli*. The growth of the organism control in the plate with agar prevailed stable and coliforms samples had the same growth in score of CFU/mL, meaning that also remained stable. These data suggest that the plasmids have origin of replication independent and can be manipulated as biological vectors (Fig. 2).

Experimental results, showed that the plasmids isolated in this article are stable by generations. Associated at that information, is interesting to know if the bacteria are able of transfer the plasmidial DNA to others species or others bacterial genders. To answer this question, was investigated the ability of bacteria grow in the trans conjugants and was analyzed too the presence and the absence of ampicillin. The results obtained (data not shown) revealed that the conjugation plasmidial was efficient. Similar the our study, a work developed in samples of pond water of ducks, RS, found that *Escherichia coli* was able of to transfer the *tetB* gene and *tetA* gene to another recipient bacteria [25].

## Concluding remarks

Taking in consideration the data of contamination found in this study and the impact of the microorganisms on public health, it’s suggested that the surfaces of the troughs are disinfected with alcohol as a method cheaper, accessible, but also a rapid bactericidal which decontaminates bacillus of tuberculosis, fungi and viruses [26]. For microbial decontamination also suggests the use of hypochlorite, sodium or calcium due to the broad spectrum of activity, with low cost and quick action.
